# Efficacy of covered and bare stent in TIPS for cirrhotic portal hypertension: A single-center randomized trial

**DOI:** 10.1038/srep21011

**Published:** 2016-02-15

**Authors:** Lei Wang, Zhibo Xiao, Zhendong Yue, Hongwei Zhao, Zhenhua Fan, Mengfei Zhao, Fuliang He, Shan Dai, Bin Qiu, Jiannan Yao, Qiushi Lin, Xiaoqun Dong, Fuquan Liu

**Affiliations:** 1 Department of Interventional Therapy, Beijing Shijitan Hospital, Capital Medical University, P.R., China; 2 Department of Plastic Surgery, The Second Affiliated Hospital of Harbin Medical University, P.R., China; 3 Chaoyang Hospital, Capital Medical University, P.R., China; 4 Department of Gastroenterology, Stephenson Cancer Center, Department of Internal Medicine, College of Medicine, The University of Oklahoma Health Sciences Center, USA

## Abstract

We conducted a single-center randomized trial to compare the efficacy of 8 mm Fluency covered stent and bare stent in transjugular intrahepatic portosystemic shunt (TIPS) for cirrhotic portal hypertension. From January 2006 to December 2010, the covered (experimental group) or bare stent (control group) was used in 131 and 127 patients, respectively. The recurrence rates of gastrointestinal bleeding (18.3% vs. 33.9%, *P* = 0.004) and refractory hydrothorax/ascites (6.9% vs. 16.5%, *P* = 0.019) in the experimental group were significantly lower than those in the control group. The cumulative restenosis rates in 1, 2, 3, 4, and 5-years in the experimental group (6.9%, 11.5%, 19.1%, 26.0%, and 35.9%, respectively) were significantly lower (*P* < 0.001) than those in the control group (27.6%, 37.0%, 49.6%, 59.8%, 74.8%, respectively). Importantly, the 4 and 5-year survival rates in the experimental group (83.2% and 76.3%, respectively) were significantly higher (*P* = 0.001 and 0.02) than those in the control group (71.7% and 62.2%, respectively). The rate of secondary interventional therapy in the experimental group was significantly lower than that in the control group (20.6% vs. 49.6%; *P* < 0.001). Therefore, Fluency covered stent has advantages over the bare stent in terms of reducing the restenosis, recurrence, and secondary interventional therapy, whereas improving the long-term survival for post-TIPS patients.

Clinical practice in the last 3 decades has demonstrated that transjugular intrahepatic portosystemic shunt (TIPS) could effectively reduce the portal venous pressure and thus prevent the complications such as uncontrollable gastrointestinal bleeding, repeated bleeding, refractory ascites, Budd-Chiari syndrome, portal vein thrombogenesis, hepatic hydrothorax, hepatopulmonary syndrome, hepatorenal syndrome, and other serious pre-operative complications before cirrhosis operation, which are caused by cirrhotic portal hypertension^1^,^2^,^3^,^4^,^5^,^6^,^7^,^8^. Large-scaled studies have reported that the success rate of TIPS can reach as high as 98.9%^9^. However, the consequently high rate of shunt channel stenosis^10^,^11^ increased the relapse of symptoms, thus severely affected the long-term efficacies, and limited the technology from widespread application.

Evidence in the past decade indicated that comparing with bare stents, covered stents could better prevent the rate of shunt channel from restenosis^12^; however, a very few studies that investigated their clinical efficacies and evaluated overall survival of the patients have reported controversial results. Thus well-designed randomized controlled trials in a large sample size along with a long-term follow up are urgently needed.

Worrt covered stent (not available in Chinese market) is specifically designed for TIPS. Previous studies have proposed that using Worrt covered stent could effectively prevent restenosis of the established shunt by TIPS, and the short-term efficacy has been evaluated^13^. In contrast, the studies on other covered stents are limited. Recently, Fluency covered stent was shown to effectively reduce portal hypertension and the complications^14^, however, evidence-based large-scaled studies are required to verify the long-term efficacies. Therefore, a prospective, single-center randomized controlled trial was performed between January 2006 and December 2010 to compare the efficacies of 8 mm covered stent and 8 mm bare stent in 839 cases who underwent TIPS at Beijing Shijitan Hospital (as shown in Fig. 1). Among them, 258 patients with portal hypertension were treated with Fluency covered stent or bare stent.

## Results

**Figure 1:**
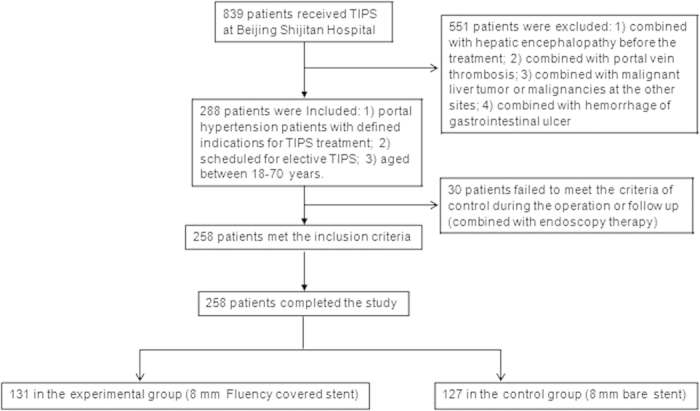
Inclusion and exclusion criteria for patient recruitment in this retrospective study.

There were 288 patients who met the inclusion criteria (with 144 in the experimental and control group each), however, 30 patients failed to meet the criteria of control during the operation or follow up. Finally, 258 patients (131 in the experimental group and 127 in the control group) completed the study (Fig.1).

### Perioperative information

**Figure 2:**
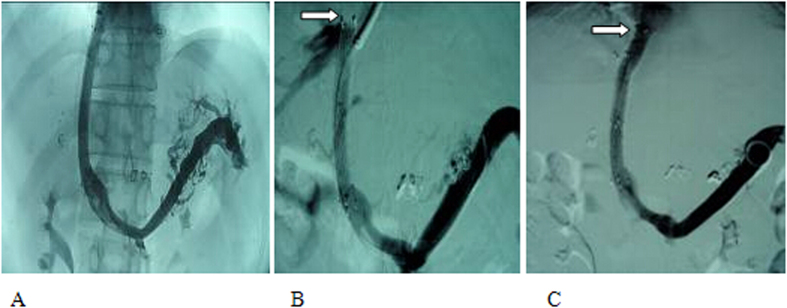
(**A**) A covered stent with the diameter of 8 × 8 mm^2^ was used to establish the shunt channel in TIPS. Portography indicated normal blood flow in the shunt channel. (**B**) Portography at 21 months after the operation identified occlusion of the shunt at the proximal (hepatic vein) end. (**C**) Another stent was implanted at the occlusion end. Portography demonstrated that the shunt channel was patent again, and the blood flow was normal.

A representative peri-operative image from patient #1 was shown in Fig. 2A. The subjects in the experimental and control group were matched by age and gender (Table 1). The portal venous pressure decreased significantly after the operation in the experimental (from 3.64 ± 0.83 to 2.34 ± 0.38 kPa; *t* = 16.30, *P* < 0.001) and the control group (from 3.77 ± 0.59 to 2.51 ± 0.26 kPa; *t* = 22.16, *p* < 0.001). The decrease of the portal venous pressure between the two groups was similar (χ^2^ = 0.935, *p* > 0.05).

### Follow up

All the 258 patients were followed up after the operation for 5 years. The percentage of gastrointestinal bleeding before the operation was similar between the experimental (93.9%; 123/131) and control (96.1%; 122/127) group (χ^2^ = 0.635, *P* = 0.426); whereas the recurrence rate of gastrointestinal bleeding (or newly diagnosed bleeding) during the follow up was significantly lower in the experimental group [18.3% (24/131) vs. 33.9% (43/127); χ^2^ = 8.098, *P* = 0.004].

The percentage of refractory hydrothorax/ascites before the operation was comparable between the experimental (15.3%, 20/131) and control (17.3%, 22/127) group (χ^2^ = 0.200, *P* = 0.655); whereas the recurrence rate of refractory hydrothorax/ascites (including non-responders, recurrent, and newly developed cases) during the follow up was significantly lower in the experimental group [6.9% (9/131) vs. 16.5% (21/127); χ^2^ = 5.547, *P* = 0.019].

The incidence rate of hepatic encephalopathy was similar between the two groups [31.3% (41/131) vs. 28.3% (36/127); χ^2^ = 0.268, *P* = 0.6055].

The rate of secondary interventional therapy in the experimental group was significantly lower than that in the control group [20.6% (27/131) vs. 49.6% (63/127); χ^2^ = 15.376, *p* < 0.001]. Moreover, 74.0% (97/131), 17.6% (23/131), and 8.4% (11/131) of the patients in the experimental group underwent 1, 2, and 3 times of interventional therapy; while the percentage in the control group was 50.4% (64/127), 25.2% (32/127), and 24.4% (31/127), respectively (Table 2).

**Figure 3:**
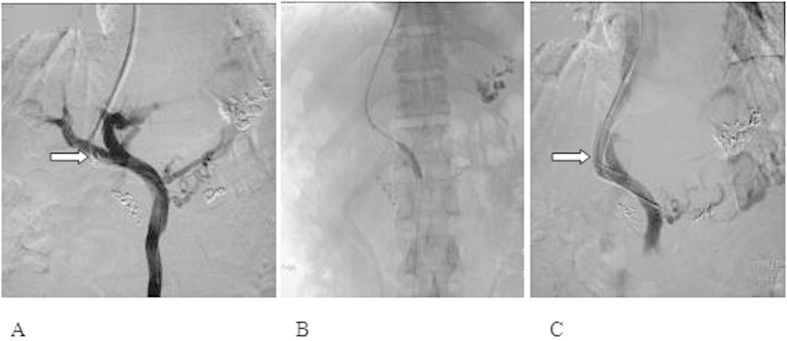
(**A**) Portography at 9 months after establishing shunt channel with 8 × 8 mm^2^ bare stent in TIPS suggested that the whole shunt channel was occluded. (**B**) Balloon dilation of the occluded shunt channel. (**C**) Another stent was implanted in the shunt channel. Portography clarified an effective shunting characterized by recovery of blood flows through the shunt channel.

As shown in Fig. 2B, when portal venous pressure increased or stenosis/occlusion of the shunt channel was identified, balloon dilation of the shunt channel and re-stenting was employed (Fig. 2C). Another representative peri-operative image from patient #2 was shown in Fig. 3A. Based on its pathological characteristics (Fig. 3B), corresponding surgical procedure (Fig. 3C) was performed. The cumulative restenosis rate in 1, 2, 3, 4, or 5-year was significantly lower in the experimental group [6.9% (9/131), 11.5% (15/131), 19.1% (25/131), 26.0% (34/131), and 35.9% (47/131), respectively] compared to the control group [27.6% (35/127), 37.0% (47/127), 49.6% (63/127), 59.8% (76/127), and 74.8% (95/127), respectively; *P* < 0.001].

The 1, 2, and 3-year survival rate was similar in the experimental [97.7% (128/131), 92.4% (121/131), and 88.5% (116/131), respectively] and control group [96.1% (122/127), 85.8% (109/127), and 80.3% (102/127), respectively; *p* > 0.05]. However, the 4 and 5-year survival rate was significantly higher in the experimental group [83.2% (109/131) vs. 71.7% (91/127)] than the control [76.3% (100/131) vs. 62.2% (79/127); *p* = 0.001 and 0.02, respectively] (Table 3).

**Figure 4:**
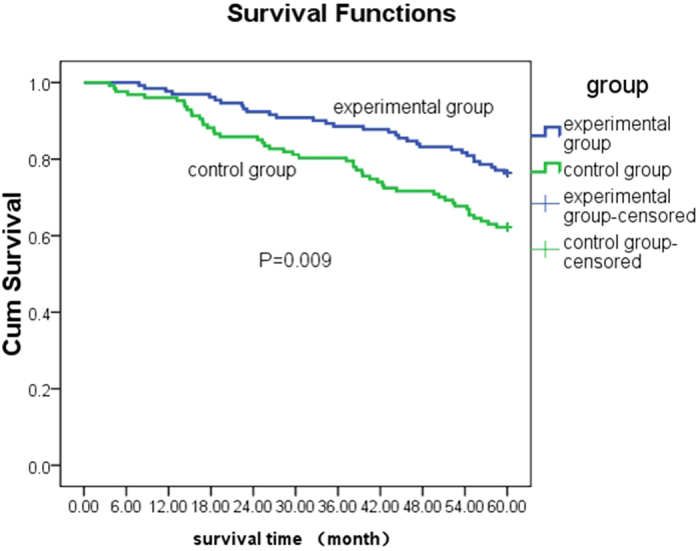
Effects of covered (experimental) vs. bare (control) stent in TIPS on overall survival (5-years follow-up) of patients with cirrhotic portal hypertension.

As shown in Fig. 4, covered stent (experimental) in TIPS exerted a significant protective effect on patients’ long-term overall survival (with 5-years follow up) compared to the bare stent (control) (log-rank test, *p* = 0.009). The median survival time of the experimental group was prolonged than the control group.

## Discussion

The short-term efficacy of TIPS in treating the complications caused by portal hypertension has been widely acknowledged^15^,^16^,^17^,^18^,^19^,^20^,^21^,^22^,^23^. However, the long-term efficacy of TIPS is restricted due to the high incidence of shunt channel restenosis and hepatic encephalopathy^24^. We compared our results with other reported studies as shown in Table 4. Shunt channel restenosis could induce the recurrence of gastrointestinal bleeding or refractory ascites, and thus further affect the survival time. Application of covered stents could significantly reduce the incidence of shunt channel restenosis. A multicenter prospective study on 114 patients receiving Viatorr covered stents found that restenosis rate at 6, 12, and 24 months was 8.1%, 20.1%, and 24.1%, respectively^12^. Another study showed that the restenosis rate in 1, 2, or 3-years after applying polytetrafluoroethylene (PTFE) covered stents was 10%, 16%, and 26%, respectively^25^. In a retrospective study^26^ conducted by Luca *et al.* included 70 patients with portal hypertension who underwent TIPS. The restenosis rate in 12- and 24-months was 38% and 21%, respectively, for 57 patients using covered stents (Viatorr; W.L. Gore & Associates, Flagstaff, Arizona, USA) vs. 85% and 29%, respectively, for 13 patients using bare stents (Wallstent endoprostheses; Boston Scientific). In a study performed by Sommer *et al.*^27^, 116 patients used bare stents (BMS; Cordis, Miami, USA) and 58 used covered stents (Viatorr) in the TIPS, and the 12-month shunt channel restenosis rate was 56.1% and 37.6%, respectively. Bureau^28^ *et al.* reported the primary patency rate of 76% and 36%, respectively (*p* = 0.001); clinical relapse recurrence rate of 10% and 29%, respectively (*p* < 0.05); free of encephalopathy rate of 67% and 51% (*p* < 0.05), respectively; and 2-year survival rate of 58% and 45%, respectively, for bare stents and Fluency covered stents. The 2-year patency rate of 76% and 36% (*p* < 0.001), clinical symptom recurrence rate of 10% and 29% (*p* < 0.05), and mortality rate of 58% and 45% (*p* < 0.05) for bare stents and Fluency covered stents, respectively; suggesting that Fluency covered stents could increase the long-term patency rate without affecting the mortality or incidence of hepatic encephalopathy and liver failure, and thus increase the middle- and long-term efficacy of TIPS.

In the present study, the cumulative restenosis rate in 1, 2, 3, 4, or 5-years in the experimental group was significantly lower than that in the control group, suggesting that covered stent can reduce both the short- and long-term restenosis rate compared to the bare stent. The recurrence rate of repeated gastrointestinal bleeding and refractory hydrothorax and ascites was significantly lower in the covered stent group than in the bare stent group. Notably, the 4- and 5- year survival rate was also higher in the covered stent group than in the bare stent group. These results were in accordance with other reports, especially the one using Viatorr stent specific for TIPS. Compared to the previous findings, the longer follow up time and the randomized controlled trial design of the present study made the results more convincing and with better comparability.

Viatorr stent applied 3 layers of PTFE with different pore-diameters of specifically processed degradations, which could completely block the leakage of bile into the stent lumen, prevent liver tissues from growing into the stent mesh, effectively cover the hepatic vein at the puncture site, and thus reduce the postoperative restenosis. Viatorr stent consists of two parts: a self-expandable metallic stent (a 2 cm bare stent for the implantation in the portal vein) and a PTFE-covered stent. A metal ring, which can be observed in imaging, is used to separate the covered and uncovered parts to help intraoperative positioning. Fluency covered stent, like the Viatorr stent also used PTFE. Two layers of PTEF cover are used in Fluency stent; the inner layer consists carbon to prevent the aggregation of platelet. Although the cover is not exactly as the same as that in Viatorr stent, the function of preventing shunt channel restenosis is similar; furthermore, the short-term efficacy of Fluency covered stent are similar to Viatorr stent, according to large-scaled international studies^13^,^14^. The findings of the present study found that the long-term survival rate in the Fluency covered stent group was significantly higher than that in the bare stent group.

Another important factor that could affect the long-term efficacy of TIPS is the development of postoperative hepatic encephalopathy. Current opinions indicate that the mechanism involved in this complication is central nervous system dysfunction caused by shunting induced or aggravated increase of nitrogen substances (e.g., blood NH3/N H4^+^). The development of hepatic encephalopathy is closely associated with the diameter of the shunt; in another word, the diameter of the shunt may determine the susceptibility to hepatic encephalopathy. A large diameter may lead to increased shunt volume, and the nitrogen substances may reduce the tolerance capacity of the central nervous system and thus induce hepatic encephalopathy^29^,^30^,^31^,^32^,^33^,^34^,^35^,^36^,^37^,^38^,^39^. Sarfeh^40^,^41^ and Rypinh^42^ also demonstrated that higher shunt volume in H-shaped shunt could increase the risk of hepatic encephalopathy. However, the incidence rate of hepatic encephalopathy after TIPS using covered stents ranged greatly (from 14.1% to 47.1%) among different studies^43^,^44^,^45^,^46^. Several studies showed that the incidence rate of hepatic encephalopathy in bare stents and 10 mm Fluency covered stents was similar (both of 20–30%); while the incidence rate of hepatic encephalopathy in 8 mm Fluency covered stent was only about 5–10%, suggesting that 8 mm Fluency covered stents could achieve effective shunt and reduce the incidence of hepatic encephalopathy^47^. Many studies have suggested that applying covered stents did not significantly reduce the incidence of hepatic encephalopathy, which is still one of the most important causes affecting the life of quality and overall survival of the patients after TIPS, which also brings heavy economic burden for the families. In the present study, we found that the incidence of hepatic encephalopathy was similar between the two groups, suggesting that using covered stents did not reduce, but slightly increased the incidence of hepatic encephalopathy as comparing with the bare stent group. We speculated that several factors including liver function, underlying diseases, and the size of the liver could be associated with this observation. A low protein diet, lactulose oral solution and dietary fiber can accelerate intestinal peristalsis and the excretion of the stool and toxic substances. All these strategies may reduce the incidence of hepatic encephalopathy.

In summary, Fluency covered stents could significantly reduce the short- and long-term rate of shunt channel restenosis and the recurrence rate of clinical symptoms, and increase the long-term survival of the patients; however, no evidence of reducing the incidence of hepatic encephalopathy was found in the present study.

## Patients and Methods

### Selection of the patients

This study has been approved by Institutional Review Board (IRB) committee at Beijing Shijitan Hospital. Informed consent was acquired from each participate before the operation. All procedures were conducted according to the guidelines approved by the ethics committee at Beijing Shijitan Hospital. This clinical trial has been public on 09/01/2015 in a publically accessible primary register that participates in the WHO International Clinical Trial Registry Platform, with the registration number of NCT02540382 (www.clinicaltrials.gov).

Inclusion and exclusion criteria were carefully designed to exclude the confounding factors. The core item in the inclusion criteria was to minimize the diameter of the shunt channel (8 mm). The inclusion criteria included: 1) portal hypertension patients with defined indications for TIPS treatment; 2) scheduled for elective TIPS; and 3) aged between 18–70 years.

The patients with one or more of the following characteristics were excluded: 1) combined with hepatic encephalopathy before the treatment; 2) combined with portal vein thrombosis; 3) combined with malignant liver tumor or malignancies at the other sites; or 4) combined with hemorrhage of gastrointestinal ulcer.

The general principle of the exclusion criteria was to exclude all the potential factors that could affect the results; for instance, for cases underwent emergent TIPS, the preoperative examination might not reflect the actual conditions of the patients. If the adjustments of the severely abnormal parameters could not be performed, then the stents could not be randomly selected, so these patients were excluded from the present study. Age (<18 or >70 years), the existence of preoperative hepatic encephalopathy, portal vein thrombosis, malignancies, as well as gastrointestinal bleeding could affect the results. In addition, several patients who met the inclusion criteria, but did not accept the randomly assigned stents, or did not establish the shunt channel as expected due to specific conditions in the operation were also excluded. For instance, the patients who were scheduled to use bare stents but received covered stents to prevent major abdominal bleeding, received both covered and bare stents, or a stent nested in the 8 mm stent were excluded. The patients who were lost to follow up or not followed up as scheduled, underwent liver transplantation, developed liver cancer or other malignancies after the operation, or died of unrelated diseases were excluded as censored cases to ensure the accuracy, randomness, and controllability of the present study.

### Sample size

Based on an expected incidence of the primary endpoint (survival rate) of about 60% at 5 years in the control group, we calculated that we would need at least 100 primary endpoint events (deaths) and a sample size of at least 250 patients to obtain 85% power to detect a significant difference between experimental and control, corresponding to a 10% reduction of relative risk (with a 2-sided type 1 error of 5%).

### Clinical characteristics

From January 2006 to December 2010, a total of 839 patients received TIPS at Beijing Shijitan Hospital. After excluding the censored cases (during the operation and follow up), 258 patients (131 in the experimental group and 127 in the control group) met the inclusion criteria and completed the clinical trial. Of the 258 included patients, there were 164 males and 94 females, with mean age of 46.3 years (range 27–69 years). Among them, 201 patients were diagnosed with post-hepatitis B cirrhosis (PHBC), 5 with post-hepatitis C cirrhosis (PHCC), 15 with alcoholic cirrhosis, 2 with primary biliary cirrhosis (PBC), 3 with autoimmune cirrhosis, 19 with Charcot syndrome, 6 with hepatic venular occlusive disease (HVOD), 1 with chronic renal insufficiency accompanying portal hypertension, and 6 with unexplained cirrhotic portal hypertension. Preoperative Child-P staging showed that 26.0% (67/258), 46.1% (119/258), and 27.9% (72/258) of the patients were in stage A, B, and C, respectively. Of the 258 patients, splenectomy and devascularization had already been performed in 29 patients, and gastroscopic sclerotherapy in 64 patients. Two hundred and forty-five patients suffered from gastrointestinal bleeding, and 42 suffered from refractory hydrothorax and ascites (29 cases were accompanied with gastrointestinal bleeding).

### Randomization

Each patient was assigned with an ID from 1 to 288 in order on admission, then a random number generator was used to randomly divide the 288 patients into group 0 or 1, with 144 patients in each. The random numbers were ordered from small to large to generate a random code. Patients with odd random codes were assigned to receive the treatments with 8 mm covered stents (experimental group), while the ones with even random codes receive the treatments with 8 mm bare stents (control group). The results of randomization were sealed in envelops, which were allocated to the clinicians in charge when the trial began. This was a double-blinded trial. Patients and physicians allocated to the intervention group were unaware of the allocated arm, outcome assessors and data analysts were kept blinded to the allocation.

### Operation procedures

#### Preoperative preparation

Liver function, coagulation, blood routine, blood type, electrocardiogram, computed tomography (CT), magnetic resonance imaging (MRI), color Doppler ultrasonography, gastroscopy, esophagography and other etiology-specific examinations were performed before the operation to exclude the bleeding caused by ulcer or other diseases. Patients’ coagulation and platelet counts were regulated to meet the requirements of TIPS. The patients and the families were informed the risks and potential consequences of the operation, and operation agreements were signed.

#### Major operation processes

Jugular vein puncture and catheterization were performed. RUPS-100 (COOK Company) sheath was delivered into the angiographic catheter to display the hepatic vein and inferior vena cava. The appropriate site was selected on the hepatic vein or inferior vena cava for the puncture of the portal vein at appropriate angle. Contrast media was injected to ensure the puncture of the portal vein was successful. The sheath was then delivered into the portal vein. When the deliver was difficult, balloon dilation was performed after portography and then the stent was implanted. Pigtail catheter was used for portography, portal venous pressure was measured, and then the varicosed vein was embolized before (or after) shunting. The portal venous pressure was measured for the second time before an 8 mm balloon was used to dilate the shunt channel and an 8 mm stent was implanted. The portal venous pressure was measured for the third time along with portography. Finally, 131 patients underwent stenting with covered stents (Bard, Fluency) and 127 patients received bare stents (EV3, protégé; Cordis, Smart).

#### Postoperative observation and treatments

All the patients were asked to rest in bed for 24 hours after the operation; pressure dressing or sandbag pressing was used, and the vital signs were monitored. In addition, antibiotics were used prophylactically. Low molecular heparin (5000 IU, twice per day) was subcutaneously injected for 5 days, and then switched to warfarin for at least 1 year. The coagulation of each patient was examined every half month to ensure the international normalized ratio (INR) was between 2 and 3. Intravenous injection of branched chain amino acid and oral administration of lactulose were also applied to prevent hepatic encephalopathy. Liver protective strategy was taken for all the patients. For the patients with sepsis, treatments based on the results of blood culture and drug sensitive test were performed in time.

#### Follow up

For each patient, systemic examinations were performed at 3- and 6-months after the operation, and then re-examinations at every 6 months. Detailed medical history and symptoms were recorded. Examinations included liver function, coagulation, blood ammonia, blood routine, color ultrasonography, esophagography, CT, and gastroscopy. When color ultrasonography suggested stenosis of the shunt channel, aggravation of varicosity, or accompanied with gastrointestinal bleeding, refractory hydrothorax or ascites, imaging of the shunt channel would be repeated, and the portal venous pressure measured. If the blood flow in the shunt channel was normal, whereas the portal venous pressure increased or stenosis/occlusion of the shunt channel was identified, balloon dilation of the shunt channel and re-stenting was performed. When the shunt volume was insufficient or the patency of the shunt channel was difficult to resume, secondary TIPS was performed to establish a second shunt channel.

### Statistical analyses

Statistical analysis was performed using SPSS software (version 17.0). Quantitative data were described with means and standard divisions, and compared by paired *t*-test. Qualitative data were compared by χ^2^ test or Fisher’s exact test. The difference in overall survival time between the control and experimental group was assessed using the log-rank test. The Kaplan–Meier method was used for calculating cumulative survival rate and survival curves. A *P* < 0.05 was considered statistically significant.

## Additional Information

**How to cite this article**: Wang, L. *et al.* Efficacy of covered and bare stent in TIPS for cirrhotic portal hypertension: A single-center randomized trial. *Sci. Rep.* **6**, 21011; doi: 10.1038/srep21011 (2016).

## Figures and Tables

**Table 1:** Preoperative characteristics of the patients.

Index	Experimental group (n = 131)	Control group (n = 127)	T/χ^2^	*P*
Gender (Male/Female)	88/43	76/51	1.497	0.221
Age (Mean ± D, years)	45.4 ± 7.0	46.7 ± 5.0	1.712	0.088
Posthepatitic cirrhosis/other cirrhosis	104/27	102/25	0.034	0.853
Child-Pugh stage (n, %)			1.278	0.528
Stage A	38 (29.0)	29 (22.8)		
Stage B	58 (44.3)	61 (48.0)		
Stage C	35 (26.7)	37 (29.2)		
Child-Pugh score	6.98 ± 1.4	7.10 ± 1.8	0.599	0.55
Gastrointestinal bleeding (Yes/No)	123/8	122/5	0.635	0.426
Refractory ascites (Yes/No)	20/111	22/105	0.2	0.655
Platelet count (×10^9)	55 ± 21.7	58 ± 29.5	0.932	0.352
Previous splenectomy and devascularization (Yes/No)	16/115	13/114	0.253	0.615
Previous sclerotherapy (Yes/No)	36/95	28/99	1.021	0.312

**Table 2:** Recurrence rate of gastrointestinal bleeding and refractory hydrothorax/ascites, incidence rate of hepatic encephalopathy and secondary interventional therapy.

Index	Experimental group (n = 131)	Control group (n = 127)	T/χ^2^	*P*
Recurrence rate of gastrointestinal bleeding (%)	18.3	33.9	8.098	0.004
Recurrence rate of refractory hydrothorax/ascites (%)	6.9	16.5	5.547	0.019
Incidence rate of hepatic encephalopathy (%)	31.3	28.3	0.268	0.6055
Rate of secondary interventional therapy (%)	20.6	49.6	15.376	<0.001

**Table 3:** Restenosis and survival rate of the patients during follow-up.

Index	1-year	2-year	3-year	4-year	5-year
Restenosis rate in the experimental group (%)	6.9	11.5	19.1	26.0	35.9
Restenosis rate in the control group (%)	27.6	37.0	49.6	59.8	74.8
χ^2^	19.512	23.072	26.730	30.279	39.483
*P*	<0.001	<0.001	<0.001	<0.001	<0.001
Survival rate in the experimental group (%)	97.7	92.4	88.5	83.2	76.3
Survival rate in the control group (%)	96.1	85.8	80.3	71.7	62.2
χ^2^	2.183	2.850	3.338	4.938	6.061
*P*	0.140	0.091	0.068	0.001	0.02

**Table 4:** Representative studies on the efficacy evaluation of stents in TIPS.

Study	Primary patency rate (%)		Encephalopathy rate (%)		Survival rate (%)	
	Bare stent	Covered stent	Bare stent	Covered stent	Bare stent	Covered stent
Vignali C *et al.*	–	79.9	23.7	–	59.3	–
Rössle M *et al.*	–	90	–	–	–	–
Luca *et al.*	62	79	32	–	81	–
Sommer *et al.*	44.9	63.4	36.5	37.5	79.1	75.6
Bureau *et al.*	36	76	33	49	69.9	54.7
Present study	27.6	6.9	28.3	31.3	80.3	88.5
